# Participant engagement in a national longitudinal study of COVID-19: Insights from the INSPIRE study

**DOI:** 10.1371/journal.pone.0325948

**Published:** 2025-07-22

**Authors:** Kris Pui Kwan Ma, Tracy Stober, Michael Gottlieb, Rachel E. Geyer, Kristin Rising, Sharon Saydah, Michelle Santangelo, Kristyn Gatling, Dylan Grau, Ralph C. Wang, Juan Carlos Montoy, Ahamed Idris, Samuel MacDonald, Mandy J. Hill, Ryan Huebinger, Maria G. Prado, Nicole L. Gentile, Erica Spatz, Caitlin Maliki, Jocelyn Dorney, Joann G. Elmore, Michelle L’Hommedieu, Robert A. Weinstein, Arjun K. Venkatesh, Kari A. Stephens, on behalf of the INSPIRE Group

**Affiliations:** 1 Department of Family Medicine, University of Washington, Seattle, Washington, United States of America; 2 Department of Emergency Medicine, University of Washington, Seattle, Washington, United States of America; 3 Department of Emergency Medicine, Rush University Medical Center, Chicago, Illinois, United States of America; 4 Department of Emergency Medicine, Sidney Kimmel Medical College, Thomas Jefferson University, Philadelphia, Pennsylvania, United States of America; 5 National Center for Immunizations and Respiratory Diseases, Centers for Disease Control and Prevention, Atlanta, Georgia, United States of America; 6 Department of Medicine, Division of Infectious Diseases, Rush University Medical Center, Chicago, Illinois, United States of America; 7 Department of Emergency Medicine, University of California, San Francisco, California, United States of America; 8 Department of Emergency Medicine, University of Texas Southwestern Medical Center, Dallas, Texas, United States of America; 9 Department of Emergency Medicine, University of Texas Health Science Center at Houston, McGovern Medical School, Houston, Texas, United States of America; 10 Department of Laboratory Medicine & Pathology, University of Washington, Seattle, Washington, United States of America; 11 Department of Epidemiology, Yale School of Public Health, New Haven, Connecticut, United States of America; 12 Section of Cardiovascular Medicine, Yale School of Medicine, New Haven, Connecticut, United States of America; 13 Department of Emergency Medicine, Yale School of Medicine, New Haven, Connecticut, United States of America; 14 Division of General Internal Medicine and Health Services Research, David Geffen School of Medicine at UCLA, Los Angeles, California, United States of America; 15 Department of Medicine, Division of Infectious Diseases, Cook County Hospital, Chicago, Illinois, United States of America; 16 Center for Outcomes Research and Evaluation (CORE), Section of Cardiovascular Medicine, Yale School of Medicine, New Haven, Connecticut, United States of America; University of California Davis School of Medicine, UNITED STATES OF AMERICA

## Abstract

**Objective:**

To examine participants’ motivations and their experiences throughout a decentralized, longitudinal COVID-19 study in the U.S.

**Methods:**

We recruited 355 participants from the Innovative Support for Patients with SARS-CoV-2 Infections Registry (INSPIRE) between November 2022 – March 2023 to answer five qualitative survey questions anonymously. We used an inductive content analysis approach to analyze the data.

**Results:**

We identified five key themes from the analysis, which reflected participants’ a) motivations to join the study, b) study benefits, c) perceptions of survey questions, d) experiences with the research process, and e) preferences for disseminating research findings. Participants were motivated to learn with researchers about COVID-19. They expressed divided opinions about the relevance of INSPIRE research questions. They reported difficulties navigating the virtual research platform and the need for making survey participation less cognitively demanding. They sought more regular feedback on study findings.

**Conclusions:**

Our findings offered insights into incorporating decentralized participatory methods in longitudinal research, strengthening reciprocal research communications, making virtual research platforms user-friendly, and employing strategies to reduce participants’ cognitive burden in research.

**Policy Implications:**

Longitudinal studies should focus on optimizing these aspects of participant engagement to produce rigorous findings that inform policy and practice on lasting effects of COVID-19 including Long COVID.

## Introduction

Patient and public engagement is valued as a core practice in public health research that informs interventions and policies to improve people’s lives [1]. Literature has shown how engaging patients and impacted communities as partners in planning, conducting, and disseminating research can enhance the quality and relevance of research [2]. To build meaningful partnerships, research teams, patients and patient organizations, funders and research institutions need to work together to design and implement research in a way that is conducive to participant engagement [3].

The COVID-19 pandemic and the emergence of Long COVID present both unique challenges and exceptional opportunities for strengthening participant engagement and partnership in research. They represented a public health crisis where an unprecedented number of people were infected with a rapidly evolving virus that was in its infancy of definition and biomedical understanding and made patient experience crucial to inform research and science [4]. Yet, the pandemic disruptions and social distancing measures made conducting in-person engaged research difficult, forcing researchers to pivot to alternative, decentralized, remote approaches for access and engagement with impacted individuals [5]. As researchers study the long-term effects of SARS-CoV-2 infection and preparedness for future pandemics, there is a need to gather insights and lessons on how to advance the science and practice of engagement using these new approaches.

Longitudinal cohort studies are key to understanding the lasting effects of SARS-CoV-2 infection, including Long COVID – its clinical signs, underlying pathophysiology, risk factors and clinical course – by tracking and monitoring symptom evolution experienced by individuals after acute SARS-CoV-2 infection [6–8]. As an example, one meta-analysis reported that almost 45% of those infected with the SARS-CoV-2 virus in the U.S. experienced at least one Long COVID symptom [9]. Those living with Long COVID experience multiple health problems (e.g., respiratory illness, cardiovascular disease, pain, fatigue, brain fog, psychosis) that could be debilitating and dangerous to one’s life [10,11]. The prevalence and significant health challenges associated with Long COVID underscore the need for longitudinal studies with patient input to advance knowledge on Long COVID.

Engaging patients with lived experience of SARS-CoV-2 infections and Long COVID in the design and conduct of longitudinal research is immensely valuable to frame research questions that resonate with participants’ priorities and needs, make surveys more acceptable for uptake by participants, and aid in contextualizing research communications to participants [12,13]. Yet consistently obtaining meaningful responses via participants’ self-reports in such studies can be challenging due to factors related to the study (e.g., length of follow-up, perceived burden), participant (e.g., understanding of the research, trust towards research team) or logistics (e.g., time and equipment) [14–17]. Especially during the pandemic, difficulty of cohort retention has been reported, and loss to follow up can compromise the validity, reliability, and generalizability of study findings [14–16]. Barriers to participant engagement in longitudinal studies remain and data are limited on how best to optimize retention and engagement among participants in longitudinal and Long COVID research [17].

To this end, we developed an open-ended qualitative survey (N = 355) to assess U.S. participants’ motivations for joining and their experience in a national longitudinal cohort study of COVID-19 that engaged participants in decentralized, virtual research platforms. The insights gathered from participants’ own perspectives were valuable to inform approaches and strategies that optimize participant engagement in future studies.

## Methods

### Study design

This study used data collected from a qualitative survey completed by a subset of participants in the Innovative Support for Patients with SARS-CoV-2 Infections Registry (INSPIRE), a CDC-funded prospective, multicenter, longitudinal cohort study on medium and long-term sequelae of SARS-CoV-2.

### The INSPIRE study

The INSPIRE study launched within the first year of the COVID-19 pandemic (December 2020) with the goal to better understand and track symptoms longitudinally based on emerging evidence of long-lasting symptoms following acute SARS-CoV-2 infections. The study methods and protocol are published in detail elsewhere [18]. Eight universities across the United States recruited participants to join the INSPIRE study. Ethics approval was obtained at universities’ respective Institutional Review Boards prior to participant recruitment.

INSPIRE study participants were ≥18 years of age, fluent in English or Spanish, and had self-reported symptoms suggestive of acute SARS-Cov-2 infection at the time of testing. Both participants with a positive SARS-CoV-2 test (“COVID-positive”) and those with a negative SARS-CoV-2 test (“COVID-negative”) but with symptoms indicative of acute SARS-Cov-2 infection were eligible to enroll. Exclusion criteria included inability to provide consent, being lawfully imprisoned, inability of the study team to confirm the result of the index diagnostic test for SARS-CoV-2, having a previous SARS-CoV-2 infection >42 days before study enrollment, and lacking access to an internet-connected device (e.g., smartphone, tablet, computer) for electronic survey completion. Enrollment window was from December 2020 to August 2022; therefore, INSPIRE study participants were enrolled during times that were pre and post introduction of vaccines (and of therapies) [19–22]. Enrolled participants were asked to complete surveys (available in English and Spanish) quarterly asking about their symptoms for SARS-CoV-2 infection; symptoms of postinfectious syndromes; reinfection or new infection with SARS-CoV-2; patient-reported outcomes on physical, mental, cognitive and social well-being; and return to work and daily activities [18]. A total of 6,044 individuals enrolled in the beginning of the INSPIRE study.

### The qualitative survey

This study analyzed and reported data that were collected from the qualitative survey that took place at the end of the INSPIRE study. Eligible participants for the qualitative survey (available in English and Spanish) included those who were fluent in English or Spanish, remained active throughout the INSPIRE study, completed a consent addendum, and finished the final survey (N = 3,878) that took place between September 2022 – February 2023. Participants received $100 incentives for completion of the final survey, but no additional incentives were provided for their optional participation in this qualitative survey. Upon completion of their final survey, participants were provided with a REDCap link that directed them to the optional qualitative survey. The qualitative survey included five open-ended questions: 1) What do you think the study did well? 2) What do you think the study could have done better? 3) What important symptoms, problems, or other questions do you think we should have asked about in the study that we may have missed? 4) What do you hope we distribute about what we discover from the study? 5) What motivated you to join and stay in the study? To maintain anonymity, the only demographic data collected was participant’s state of residence. The survey was anonymous so that honest and open feedback could be obtained while protecting participants’ privacy. Survey responses were collected across all eight university sites between November 2022 to March 2023.

### Analysis

Survey responses were downloaded from REDCap and imported to the Dedoose software [23] for coding. Spanish responses were translated in English and back-translated for verification by two bilingual research staff. We used content analysis approach to establish and interpret meaning from textual content collected via surveys [24,25]. A flowchart of the coding process and details of the codebook are included in the S1 Fig and S1–S2 Tables. Our coding team (KM, TS, RG, MP) consisted of a qualitative research expert, a Long COVID patient advisor, research staff who led study recruitment and data collection activities, and a eneral research staff member with no prior involvement in the INSPIRE study. Given the different roles and backgrounds, the team engaged in a continuous and collaborative process of discussing their prior assumptions and emerging codes in the context of the study, which helped counter biases and expand interpretations of the findings [26]. The coding team developed codes using line-by-line reading of a subsample (20%) of survey responses. This iterative approach allowed the team to begin identifying and organizing thematic categories to develop a codebook. Then, three coders (TS, RG, MP) applied the codebook and analyzed the remaining survey responses. All survey responses (100%) were double-coded to ensure consistent application and interpretation of the codebook. The coding team met regularly throughout the coding process to resolve discrepancies, adjust code definitions, and assess saturation [27]. Comparative method was used to assess code saturation by coding relevant segments in the data and systematically comparing these codes with those already established from earlier data. Code saturation was reached when no new codes and patterns were identified in the collected data. After the coding was complete, the coders met with the senior investigators of this study (MG, KR, RAW, AKV, KAS) to interpret the codes and generate themes. All INSPIRE study investigators reached consensus on the themes reported in this paper.

## Results

Of the 3,878 INSPIRE study participants who were eligible for the qualitative survey, 355 (9.2%) completed the survey. Three responses were in Spanish. The respondents represented seven states of the eight recruiting university sites – Washington, California, Illinois, Connecticut, Texas, Pennsylvania, New Jersey, and 14 other states (see breakdown in Table 1). The results are described in five themes (see sample participant quotes in Table 2).

**Table 1:** Geographic distribution of survey participants based on state of residence.

States	N (%)
Washington	115 (32.4%)
California	64 (18.0%)
Illinois	60 (16.9%)
Connecticut	42 (11.8%)
Texas	22 (6.2%)
Pennsylvania	15 (4.2%)
New Jersey	8 (2.3%)
Other	28 (7.9%)

*Note*. One missing case was in the participant’s state of residence. Other states included Alaska, Arkansas, Florida, Georgia, Indiana, Kentucky, Maine, Maryland, Missouri, Montana, New York, Oklahoma, Oregon and Utah.

**Table 2:** Summary of Themes and Participant Quotes.

Themes and Subthemes	Participant Quotes
**Motivations to join the study**	
• Participants desired to help.	Just being helpful in this pandemic time – I felt like I could help after getting COVID, and maybe it would help in future.
• Participants wanted to share personal experience.	Weird s*** was happening to me and really impacting my life. I wanted someone to be able to learn from it to help me and others.
• Participants wanted to learn more about COVID.	Wanting to stay COVID aware and helping others learn about it.
• Participants hoped to contribute to science and research.	I’m a nurse and I believe unbiased research is very valuable.
• Participants appreciated to be compensated.	Delivered rewards in a timely manner. I think that keeps people motivated to do surveys.
**Benefits from the study**	
• Participants reported filling out surveys over time helped with symptom self-monitoring and awareness, which prompted help-seeking.	Made me aware of issues that I have that need to be addressed with my provider.
• Participants wished to receive guidance and information based on the symptoms they reported on the surveys.	[I wish the study was better at] keeping me updated and brought into new studies and debrief me about the results or findings so far and give notes once joined the study.
**Perceptions of the survey questions**	
• Participants were divided in how they perceived whether the questions aligned with their lived experience and needs.	Your surveys didn’t adequately capture the experience of someone who had COVID and completely recovered. I feel that the surveys were very detailed and covered my symptoms and concerns.
• Participants wanted space in surveys to share their personal experiences.	During each survey I found myself wishing I could explain my answers better or just tell my personal version of the illness.
• Participants suggested clarifying whether the questions being asked were due to COVID or other causes.	Specified when asking about pain if it was related to covid or not. I had a shoulder injury and was in pain. Not covid related.
• Participants found some wording and phrasing of the questions confusing.	[you could have done better with] questions especially medical explain in layman terms.
• What participants think is important to study about COVID is expansive and broader than the scope of the INSPIRE study.	Maybe [ask more] questions about social support? It’s such a safety net for chronic health issues. I hope this study helps inform future vaccination protocols, i.e., effectiveness and efficacy based on timing of vaccination, illness, etc.
**Experience with the research process**	
• Participants appreciated the convenience of online surveys and availability of human ‘touch’ and assistance from researchers.	[The study did well by] following up by a human to alert me of a missed survey (And having the same guy each time – helped personalize the experience).
• Participants suggested ways to improve survey accessibility that consider their COVID symptoms and cognitive functioning.	Please have the survey remember basic information about me (like my name and vaccinations dates) so I don’t need to re-write it every time.
• Participants thought the reminders were helpful to prompt them to complete surveys at each time point.	The reminders. Not super annoying, and I needed those, because I often received one or two in a moment where I was not able to answer, and then forgot until the next reminder.
• Participants reported difficulties in navigating multiple platforms, linking medical records to surveys, and using the gift cards.	The ‘portal’ was limited/confusing. I couldn’t see any record of completing my old surveys, etc. Essentially, everything was run via email, so, what was the point of the Hugo health portal.
**Preferences for dissemination of research findings**	
• Participants reported distrust with information in the public sphere and wanted factual, unbiased findings.	[Distribute] unbiased facts to the public, unlike the political self-serving government produced medical guidance based on biases and industry influence.
• Participants wanted actionable findings that help them to acknowledge and address Long COVID conditions.	[I hope you share] data that will help people know they are not alone and the problems that come with Long COVID can and should be taken seriously.
• Participants wanted findings to be easily understood and used by diverse audience (i.e., healthcare professionals, researchers, and general public).	Information that benefits people and communities including healthcare providers and preventative medicine around the world.

### Motivations to join the study

Analysis identified five motivations (i.e., desire to help, to share personal experience, to learn about COVID, to contribute to science, and to be compensated) shared by participants for joining the INSPIRE study. Participants reported joining the study because they “want to help”. Many had lived experience of SARS-CoV-2 infections and Long COVID symptoms and wanted to share personal experience, so that others might learn something from their experience. Participants also expressed interest in keeping abreast of COVID-19 research. Participants understood the value of science and research, given their own professions and training; thus, they contributed their data to the study. Adequate and timely compensation was also considered motivating by participants.

### Benefits from the study

Several participants spontaneously mentioned how they benefitted from joining the INSPIRE study. These participants reported that filling out the quarterly surveys over time had helped them monitor and track their own symptoms, increased their self-awareness of COVID-related concerns and conditions, and prompted them to seek timely medical help. For example, a participant said, “[Joining this study] helped me identify an ongoing tiredness issue and got it addressed!”

Participants had suggestions about how the study might benefit participants more. One suggestion was for the study team to provide information and guidance to participants based on responses to the surveys. For instance, a participant said, “If someone indicates they are depressed, would it be possible to refer them to providers to assist with that, or at least links to help them to decide what to do?” Another participant said, “I would like to know the results. When asked about Epstein-Barr and Shingles, I wonder what that has to do with this COVID study because my son has had a lot of medical issues since getting the vaccine and now has been told he has Epstein-Barr. I would like to know the connection.” Participants wished there were opportunities to debrief their survey responses with the study team.

### Perceptions of survey questions

Participants were divided in how they perceived the relevance of the longitudinal survey questions in the INSPIRE study to their lived experience. They felt the questions did not encompass the variety and the full spectrum of their lived experiences, which could include experiencing Long COVID symptoms, recovering from the acute infection, and experiencing symptoms that were suggestive of pre-existing medical conditions instead of SARS-CoV-2 infections. They also suggested clarifications for medical jargon that were used in some questions that were seen as poorly worded. They wished there were opportunities for free-text responses or other inclusive options that would allow them to describe and explain their experiences. Participants expressed that they have many COVID-related questions that they wished the study could further explore, even though some of them might be beyond the initial scope of the INSPIRE study. Some of these topics and questions included better characterization of SARS-CoV-2 infections and Long COVID symptoms, risk factors and prognoses, better treatment options, how vaccination affects symptoms, and the impact of comorbidities and pandemic stress on recovery from SARS-CoV-2 infections.

### Experience with the research process

Participants reported difficulties in navigating the research process; specifically, participants experienced issues with survey implementation (e.g., entering date fields) and technical difficulties as they were asked to use multiple digital platforms to link medical records and redeem electronic gift cards. Furthermore, the quarterly surveys required participants’ recollections and reports of their COVID testing and vaccination dates, which some participants found difficult to answer. These issues may have been compounded by known cognitive symptoms (e.g., brain fog, confusion, memory impairments) associated with SARS-CoV-2 infections and sequelae, making these tasks more difficult. Despite this, participants appreciated the surveys being online and the convenience of allowing them to easily complete the surveys from their computer or mobile device anytime and anywhere. Participants also acknowledged having a dedicated research team (“real human interaction”), that provided reminders through phone calls, emails, or SMS text messages and assisted them with any difficulties with the study, helped personalize and enhance their engagement and continued participation with the study.

### Preferences for dissemination of research findings

Participants hoped to see findings shared from this study that were actionable and tailored for a range of audiences, including healthcare professionals, public health, and the general population. Specifically, they wanted the findings to acknowledge Long COVID as a health issue and encourage people at high risk or experiencing these symptoms to seek help. They wanted to see the study findings outside of a traditional academic publishing arena and share back to the participants themselves in lay terminology. For example, they suggested holding video conferences on the results of the study or publicly distributing results via TV, radio, internet, and newspapers. Also, participants emphasized the importance of receiving information from trusted and unbiased sources. Some participants expressed their distrust to the current sources of COVID-related information in public and wanted the data they contributed to be shared in a factual, unbiased, and transparent manner.

## Discussion

We report 355 survey participants’ perspectives on their motivations and experiences of engaging in a longitudinal cohort study of COVID-19. Participant engagement in longitudinal cohort studies is not a new concept, but its application varies and barriers to implementation remain [17]. With the large number of reported Long COVID cases [9,28,29] and inadequacy of clinical records of patient histories to capture fully the dimensions of patient suffering [30], longitudinal cohort surveys play a key role in advancing the science and knowledge of the long-term effects of SARS-CoV-2 infections. The participants’ responses offered valuable insights for optimizing participant engagement and retention in future longitudinal research on Long COVID.

Participants’ motivations to join the INSPIRE study were similar to those reported in other non-COVID related studies: altruistic reasons (desire to help, contribute to science and society), reasons related to their illness (want to know about the disease), advocacy (elevate patient voices and participation in decision-making), and personal reasons (financial incentives, enjoyment) [14,31,32]. However, the combined desires to share personal experience of the illness and to quickly gather new information about the disease were particularly prominent in our data as well as in other COVID-related studies [14]. These findings suggested that in a pandemic context where people face uncertainty and ambiguity around a new and complex disease like SARS-CoV-2 infections and sequelae, participants wish to have a more active role in research where they can leverage their illness experience to illuminate scientific discoveries and keep learning about the disease as the pandemic evolves. To respond to participants’ motivations and reinforce engagement, recent cohort studies on COVID have begun embedding participatory research approaches [12,17,33,34], using novel engagement strategies such as participant involvement panels, knowledge co-production models, participant-proposed questions, and real-time feedback mechanisms [33,35]. Furthermore, it is important to note that patient-led efforts have significantly contributed to our understanding of SARS-CoV-2 infections and sequelae during the pandemic. Patient-led groups (e.g., Patient-Led Research Collaborative) have helped recognize the symptoms and patterns of Long COVID that formal studies had yet to explore and called attention to many under-researched areas that lead to new clinical trials [4,36]. Therefore, research efforts and findings led and motivated by patients with lived experience should also be amplified and incorporated by researchers, as they continue to work with patients to drive forward the science and treatment of Long COVID.

Participants’ desire for reciprocity in research partnerships with researchers are explicitly expressed across multiple themes. It is found in both our and other studies that reciprocal communications between participants and researchers could have helped respond to participants’ needs and encouraged better engagement in research. For example, INSPIRE study participants wished to receive regular updates on the findings and follow-up support (e.g., education and referral resources) on their survey responses. Future studies could consider providing this information directly or through the patient’s provider (when collaborating with healthcare systems). Although patient-specific feedback in the absence of patient physical examinations and detailed histories may not be appropriate, study teams could still provide general study updates via newsletters (which was done in INSPIRE study) or host town hall meetings or virtual conferences to answer participants’ questions [34]. Data on how participants use that information and feedback and their changes in COVID-related symptoms/knowledge/behaviors over time can be collected for analyses in addition to their survey responses [37]. Furthermore, creating opportunities for reciprocal dialogues where participants can offer input and suggestions on survey questions could be particularly crucial in the dynamic context of a pandemic, given that their issues of concern and needs were evolving over time. To address questions of unclear relevance or understanding and technical difficulties that were raised by our survey respondents, soliciting participant input in the survey design process such as organizing regular feedback sessions with patient advisory panels or distributing pre-survey questionnaires to gather input on topics of participant interest can help improve the study’s relevance to participants’ lived experience, study participation, and data quality [37].

Current findings also pointed to the need for leveraging the advantages and overcoming challenges of using a decentralized approach to longitudinal studies – a paradigm shift in research that has gained popularity during the COVID-19 pandemic [38–40]. The INSPIRE study was designed for rapid enrollment and ease of participation through prospective, decentralized, digitally enabled data collection. Various modalities of digital technology were used: electronic consent, online surveys, e-reminders, partnership with a digital health technology provider (Hugo Health) to connect medical records, and distribution of incentives on a virtual platform. INSPIRE study participants enjoyed the ease and convenience of completing the study virtually; but they struggled to navigate the digital platforms and surveys efficiently. Making digital research platforms more user-friendly, accessible, and participant-centered is necessary to advance real-world implementation of fully remote, large-scale studies [41]. Furthermore, the level of touch (i.e., direct interaction between participant and researcher) in decentralized studies is less standardized than those in traditional research, which can at times make it difficult to determine the appropriate level of touch that will optimize participant experience [41,42]. Participants in the INSPIRE study appreciated a certain level of touch where they were able to receive study reminders via phone calls or texts from consistent research staff and where they could contact staff for questions and support. Some participants also requested more direct participant-researcher interaction when it comes to following up on study updates and findings. Given the level of touch and interaction could be flexible and change depending on participants’ needs, future studies can examine how various levels of touch shape participant engagement and relationships with researchers in decentralized studies.

Employing strategies to reduce participant burden (i.e., time, effort, and cognitive resources required of participants to complete surveys) should be a priority in survey design when engaging participants with cognitive impairments related to their disease, as often experienced by people with Long COVID. As the growing body of literature shows, some patients experience cognitive impairments such as brain fog, memory decline, concentration difficulties and fatigue after SARS-CoV-2 infections, which can add difficulties to their research participation [43]. To reduce task difficulty and improve survey data quality, researchers can adapt study materials for mixed abilities and collect partial data from proxy/data linkages rather than relying heavily on participants’ recollection [44]. For instance, survey respondents suggested that data regarding the details of their COVID tests and vaccinations could be extracted from their linked medical records or from previous survey responses. Additionally, providing a save and finish later feature option allows participants to take breaks during survey participation. Since longitudinal studies that employ strategies to address participant burden showed better retention rates compared to studies that did not [44], careful consideration of participant burden through early involvement and input from participants when designing the surveys would be helpful.

The benefits perceived and experienced by participants joining the INSPIRE study alluded to the potential ‘therapeutic’ benefits of longitudinal surveys. It is notable that some participants used the repeated assessments as a symptom self-monitoring tool that helped increase their awareness and help-seeking behaviors, although this was not a planned purpose of the INSPIRE study. Prior literature on patient registries mentioned that some patients reported ‘therapeutic’ benefits when answering certain types of survey questions that were of high perceived relevance to their experience [37]. However, further evidence is needed to understand if the intriguing ’therapeutic’ benefits were truly clinically significant and whether the experience of ‘therapeutic’ benefits impact research participation or the individual outcomes and behaviors that are assessed in longitudinal health research.

### Limitations

We acknowledged two main limitations to this study. First, the data were collected only from participants who responded to the optional feedback survey after their final survey and thus are susceptible to selection bias. The response rate was low compared to those of prior surveys in the INSPIRE study due to attrition, and the respondents were limited to those who remained in the study through the final survey. It is beyond the scope of this paper to conclude the reasons for the low retention of participants in the INSPIRE study, though the qualitative findings presented here might offer some clues for further investigation. The findings were exploratory in nature and were not intended to be representative nor generalized to all participants in the INSPIRE study. The findings likely reflect the views of research participants who were motivated to engage in most INSPIRE study activities and understood the research process to provide feedback. Survey nonrespondents or individuals who previously dropped out from the INSPIRE study might have different views and experiences from those that were reported in this study. Because of the anonymous nature of the survey, we could not investigate whether participant characteristics, index COVID status, symptoms, or other factors were associated with survey participation. We did observe that the responses were predominantly reported in English, despite the availability of our survey in both English and Spanish, suggesting one type of selection bias. Future research can use random or stratified sampling to mitigate selection bias and include personalized invitations/follow-up to improve the survey response rate.

Second, because data on respondents’ characteristics were not available, it is not known whether the experiences in the study or the uptake of research activities differ among participants of different characteristics (e.g., those living with Long COVID symptoms vs. those who recovered from SARS-CoV-2 infections vs. those who were tested negative for SARS-CoV-2 infections at the time of enrollment) [45]. Also, for the purpose and design of this study, the qualitative data are intended to identify themes and patterns of the participant experiences and provide rich description and nuances of the experiences. These findings are not meant to be quantified and cannot be used in determining the magnitude or prevalence of the experiences, because quantification of such qualitative data may risk misinterpretation of the findings. Thus, future research can incorporate both qualitative and quantitative methods to investigate associations between participants’ characteristics and their study participation over time and examine nonrespondents’ perspectives and experiences of engaging in longitudinal surveys. Despite the limitations, the online qualitative feedback survey used in this study reached geographically dispersed participants on a national scale that would not be easily achieved otherwise [25]. As the number of longitudinal cohort studies on COVID continues to grow, this is one of the very few studies that gathered participants’ perspective on their study engagement, which will serve as useful feedback for other cohort studies to focus on what participants with lived experience value when participating in COVID-related cohort studies.

### Public health implications

Longitudinal studies with robust participant engagement can help inform policy and practice to address the adverse, long-term impacts of SARS-CoV-2 infections. Few studies have examined the participants’ experience of engagement in longitudinal COVID research, especially in a pandemic setting. Our findings provided rich and important insights into the why and how of optimizing participant engagement which have implications in future longitudinal COVID-19 studies and pandemic research preparedness.

## Conclusion

Participants desired stronger and closer partnerships with researchers to collaboratively construct knowledge about COVID and Long COVID. Our findings, based on participant perspectives, inform approaches that foster participation in future longitudinal studies by incorporating robust participatory research methods, maintaining a reciprocal dialogue between researchers and participants, making decentralized and virtual research platforms more user-friendly, and employing cognitive load reduction strategies in survey design to remove barriers.

## Supporting information

S1 Fig Flowchart of the Coding Process. (DOCX)

S1 Table Initial codes and codebook. (DOCX)

S2 Table Refined Codebook. (DOCX)

S1 File Authors of the INSPIRE Group. (DOCX)
