# Supplementary figures and images for: Participant engagement in a national longitudinal study of COVID-19: Insights from the INSPIRE study
Source: PLoS One. 2025 Jul 22;20(7):e0325948. doi: 10.1371/journal.pone.0325948 (PMC12282896; doi:10.1371/journal.pone.0325948)

**S1 Fig. Flowchart of the Coding Process**

**
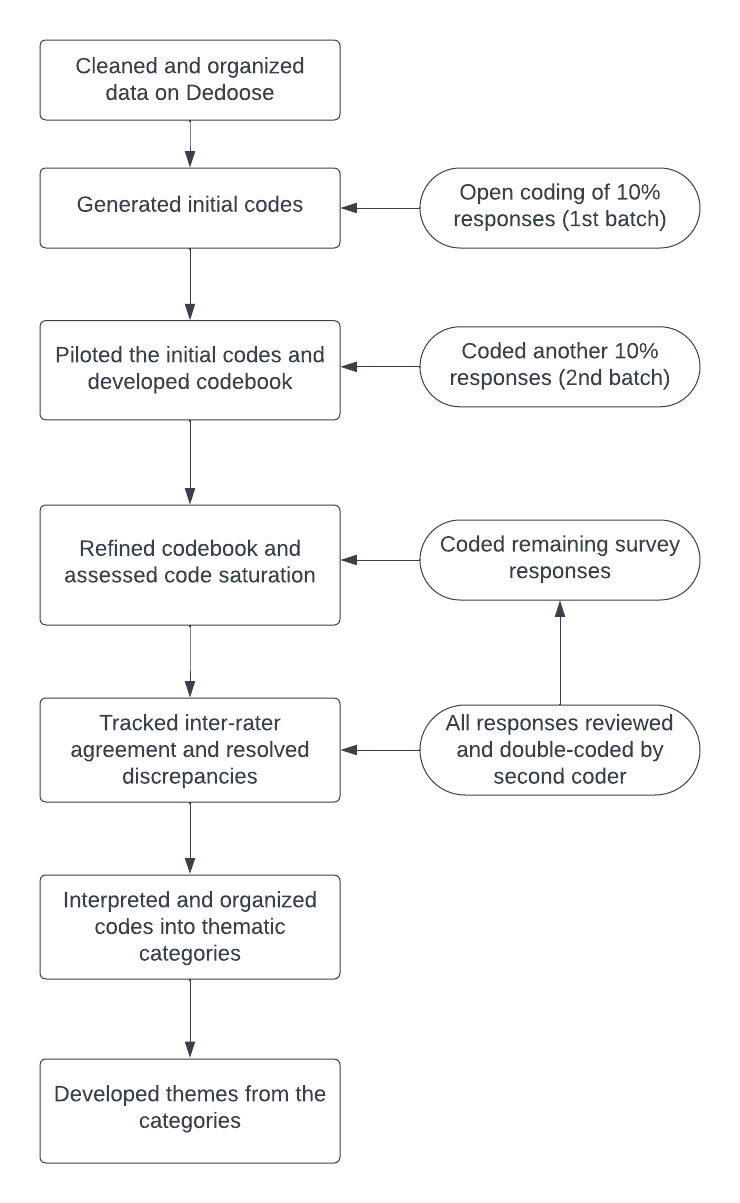
**

Supplement: S1 Fig — (DOCX) [file pone.0325948.s001.docx]
