# Supplementary material for: Participant engagement in a national longitudinal study of COVID-19: Insights from the INSPIRE study
Source: PLoS One. 2025 Jul 22;20(7):e0325948. doi: 10.1371/journal.pone.0325948 (PMC12282896; doi:10.1371/journal.pone.0325948)
Supplement: S1 Table — (DOCX) [file pone.0325948.s002.docx]

**S1 Table. Initial codes and codebook**

| *Example codes* | *Groupings* |
| --- | --- |
| Wanted to help (want to help others, want to help medicine/science, want to help because of personal experience)  Wanted to be part of COVID study  Wanted to learn more  Concerned about personal health  Compensation  Employed in sciences and appreciate how important this is  Survey is easy to complete | Motivating factors |
| Questions were on target  More detailed/open ended questions  Inapplicable questions to situation  Repetitive  Questions construction and phrasing | Types/wording of questions |
| Responsive  Follow-up reminders (emails, check in more often)  Reminders (missed survey reminders)  Provide more COVID/Long COVID information | Participant-researcher interactions |
| Connect to healthcare portal, Hugo account, link medical records  Ease of survey completion (both positive and negative feedback)  Consistency of surveys  Redemption process/logistics of gift cards | Participant experience/study-wide |
| Long-term effects of COVID/Long COVID (how to prevent, minimize, diagnose)  Vaccine  Infections and Symptoms  Treatment options  Other general information about COVID  Questions surveys did not ask (symptoms to add, pre-existing conditions, social support, personal COVID experience, symptoms before pandemic) | COVID-19 Information/Topics that participants are interested |
| Distribute results in plain language  Audience for dissemination  Disseminate study findings during study period | How and when to disseminate findings |
