# Supplementary material for: Participant engagement in a national longitudinal study of COVID-19: Insights from the INSPIRE study
Source: PLoS One. 2025 Jul 22;20(7):e0325948. doi: 10.1371/journal.pone.0325948 (PMC12282896; doi:10.1371/journal.pone.0325948)
Supplement: S2 Table — (DOCX) [file pone.0325948.s003.docx]

**S2 Table. Refined Codebook**

| *Example Codes* | *Categories* | *Themes* |
| --- | --- | --- |
| Want to help others  Want to help because of personal experience  Wanted to be part of COVID study  Wanted to learn more  Concerned about personal health  Want to help medicine/science  Be a data point  Love doing surveys  Appreciate the gift cards | Desire to help  Desire to share personal experiences  Desire to learn more about COVID  Value/interest/enjoy/contribute to science, research, medicine  Compensation | **Motivations to join the study** |
| “Made me aware of the issues that I want to talk with my provider”  Disseminate findings during study period Provide more COVID/Long COVID information | Surveys make participants aware of symptoms and prompt to seek help  Participants want feedback or follow-up from their survey responses | **Benefits from the study** |
| Questions were on target  Ask questions on recovery, not just symptoms  “Some of my symptoms are not due to COVID”  “Questions well-constructed”, “easy to read”, use lay language  “Space to write free text”  Vaccine, Long COVID symptoms, Infections, Co-occurring conditions, Treatment options, Patient experience before diagnosis, Impact of the pandemic | Relevance (content) of the questions  Clarification for questions asking COVID vs. other causes  Construction (phrasing) of the questions  Open-ended questions  Expansive list of COVID-19 topics that participants are interested in studying | **Perceptions of the survey questions** |
| Ease of survey completion (positive feedback)  Communication with researchers (easy to contact, following up)  Ease of survey completion (negative feedback or suggestions – i.e., auto-filling, color, animations)  Follow-up reminders (emails, check-ins)  Reminders (missed survey reminders)  Connect to healthcare portal, Hugo account, link medical records | Convenience of online surveys  Availability of human ‘touch’ and assistance from researchers  Survey accessibility and cognitive burden  Reminders  Technical difficulties | **Experience with the research process** |
| Distrust with the information they received  Hope to distribute unbiased facts to the public  Acknowledge Long COVID exists and encourage people to seek help  Format and language for dissemination  Audience for dissemination | Distrust  Actionable findings  Findings to be easily understood and used by diverse audiences | **Preferences for dissemination of research findings** |
