# Supplementary material for: Participant engagement in a national longitudinal study of COVID-19: Insights from the INSPIRE study
Source: PLoS One. 2025 Jul 22;20(7):e0325948. doi: 10.1371/journal.pone.0325948 (PMC12282896; doi:10.1371/journal.pone.0325948)
Supplement: S1 File — (DOCX) [file pone.0325948.s004.docx]

**S1 File. Authors of the INSPIRE Group**

INSPIRE Group

Lead contact for this group:
Robert A. Weinstein, MD, Principal Investigator, [ROBERT_WEINSTEIN@rush.edu](mailto:ROBERT_WEINSTEIN@rush.edu)
Michael Gottlieb, MD, Principal Investigator, [Michael_A_Gottlieb@rush.edu](mailto:Michael_A_Gottlieb@rush.edu)

**Rush University, Administrative Core & Enrolling Site**

**Study-wide Co- Principal Investigators:** Robert A. Weinstein, MD, Principal Investigator; Michael Gottlieb, MD, Principal Investigator

**Core research team:** Michelle Santangelo, MS, Research Manager; Katherine Koo, MS-HSM, Program Manager; Antonia Derden, BA, Administrative Assistant

**Site Investigators:** Michael Gottlieb, MD, Site Principal Investigator

**Site research team:** Kristyn Gatling, MA, Research Coordinator. Research Assistants: Zohaib Ahmed, BS; Chloe Gomez; Diego Guzman, BS; Minna Hassaballa, BA; Ryan Jerger; Amro (Marshall) Kaadan, BS

**Yale University, Analytic Core & Enrolling Site**

**Core Investigators:** Principal Investigators: Arjun Venkatesh, MD, MBA, MHS; Erica S. Spatz MD, MHS

**Core research team:** Research Managers: Jeremiah Kinsman, MPH, NREMT, Caitlin Malicki**,** MPH. Statisticians: Zhenqiu Lin, PhD; Shu-Xia Li, PhD; Huihui Yu, PhD; Imtiaz Ebna Mannan, MS; Zimo Yang, MS; Mengni Liu, MS

**Site Investigators:** Site Principal Investigators: Arjun Venkatesh, MD, MBA, MHS, Erica S. Spatz MD, MPH. Site Co-Investigator: Andrew Ulrich, MD

**Site Research team:** Research Managers: Jeremiah Kinsman, MPH, NREMT, Caitlin Malicki, MPH. Research Coordinator: Jocelyn Dorney, MPH. Research Assistants: Senyte Pierce, BA; Xavier Puente, BA; Wafa Salah, BA

**University of Washington, Clinical Core & Enrolling Site**

**Core Investigators:** Graham Nichol, MD, Principal Investigator; Kari A. Stephens PhD, MS, Principal Investigator

**Core research team:** Jill Anderson, BSN, RN, Clinical Core Program Manager; Mary Schiffgens, MBA, Grant & Finance Manager; Dana Morse, RN, BSN, Research Coordinator; Karen Adams, BA, Regulatory Specialist; Tracy Stober, BA, MA, Patient Representative; Zenoura Maat, Research Assistant

**Site Investigators:** Kelli N. O’Laughlin, MD, MPH, Site Principal Investigator; Nikki Gentile, MD, PhD, Co-Investigator

**Site research team:** Research Coordinators: Rachel E. Geyer, MPH; Michael Willis, AS, BSHS; Zihan Zhang, MS, Analyst; Gary Chang, PhD, Senior Biostatistician. Victoria Lyon, MPH, Project Manager. Research Assistants: Robin E. Klabbers, MSc in Medicine, MSc in Global Health; Luis Ruiz, BA; Kerry Malone, BA; Jasmine Park

**Thomas Jefferson University, Enrolling Site**

**Site Investigators:** Kristin Rising, MD, MS, Site Principal Investigator; Efrat Kean, MD, Co-Investigator; Anna Marie Chang, MD, MSCE

**Site research team:** Nurse Coordinator: Nicole Renzi, RN. Program Manager: Phillip Watts, BA, MM, CCRP. Research Coordinators: Morgan Kelly, BS; Kevin Schaeffer, BS; Dylan Grau, BS; David Cheng, BS; Carly Shutty, BSN; Alex Charlton, BS; Lindsey Shughart, BS; Hailey Shughart, BA, CCRP; Grace Amadio, MD, CCRP; Jessica Miao, BA. Research Assistants: Paavali Hannikainen, BS

**University of California, Los Angeles, Enrolling Site**

**Site Investigators:** Joann G. Elmore, MD, MPH, Site Principal Investigator, Lauren E. Wisk, PhD, Co-Investigator

**Site research team:** Michelle L’Hommedieu, PhD, Site Program Director; Chris Chandler, BA, Research Assistant; Megan Eguchi, MPH, Data Analyst; Kate Diaz Roldan, MPH, Research Assistant; Raul Moreno, BA, Administrative Analyst

**University of California, San Francisco, Enrolling Site**

**Site Investigators:** Robert Rodriguez, MD, Site Principal Investigator; Ralph C. Wang, MD, MAS, Site Principal Investigator; Juan Carlos Montoy, MD, PhD, Site Principal Investigator

**Site research team:** Robin Kemball, MPH, Program Manager; Research Coordinators: Virginia Chan, MPH; Cecilia Lara Chavez; Angela Wong, BA; Mireya Arreguin, BS

**University of Texas Health Science Center at Houston, Enrolling Site**

**Site Investigators:** Mandy Hill, DrPH, MPH, Site Principal Investigator; Ryan Huebinger Site, MD, Site Principal Investigator.

**Site research team**: Arun Kane, BA, Research Coordinator; Peter Nikonowicz, BA, Research Coordinator; Sarah Sapp, MPH, Research Coordinator

**University of Texas Southwestern Medical Center, Enrolling Site**

**Site Investigators:** Ahamed H. Idris, MD, Site Principal Investigator; Samuel McDonald, MD, Co-Investigator

**Site research team:** David Gallegos, BS, Research Coordinator; Katherine Riley Martin, BS, MS, Research Assistant

**Centers for Disease Control and Prevention (CDC)**

**Investigators:** Sharon Saydah, PhD; Ian D. Plumb, MBBS, MSc; Aron J. Hall, DVM, MSPH; Melissa Briggs-Hagen, MD, MPH
